# Assessment of the macrovascular contribution to resting-state fMRI functional connectivity at 3 Tesla

**DOI:** 10.1162/imag_a_00174

**Published:** 2024-05-20

**Authors:** Xiaole Z. Zhong, Yunjie Tong, J. Jean Chen

**Affiliations:** Rotman Research Institute at Baycrest, Toronto, ON, Canada; Department of Medical Biophysics, University of Toronto, Toronto, ON, Canada; Weldon School of Biomedical Engineering, Purdue University, West Lafayette, IN, United States; Department of Biomedical Engineering, University of Toronto, Toronto, ON, Canada

**Keywords:** blood-oxygenation-level dependent fMRI, macrovascular BOLD, resting-state fluctuation amplitude (RSFA), functional connectivity, extravascular BOLD

## Abstract

In resting-state functional magnetic resonance imaging (rs-fMRI) functional connectivity (FC) mapping, temporal correlation is widely assumed to reflect synchronized neural-related activity. Although a large number of studies have demonstrated the potential vascular effects on FC, little research has been conducted on FC resulting from macrovascular signal fluctuations. Previously, our study found (Tong, Yao, et al., 2019) a robust anti-correlation between the fMRI signals in the internal carotid artery and the internal jugular vein (and the sagittal sinus). The present study extends the previous study to include all detectable major veins and arteries in the brain in a systematic analysis of the macrovascular contribution to the functional connectivity of the whole-gray matter (GM). This study demonstrates that: (1) The macrovasculature consistently exhibited strong correlational connectivity among itself, with the sign of the correlations varying between arterial and venous connectivity; (2) GM connectivity was found to have a strong macrovascular contribution, stronger from veins than arteries; (3) FC originating from the macrovasculature displayed disproportionately high spatial variability compared to that associated with all GM voxels; and (4) macrovascular contributions to connectivity were still evident well beyond the confines of the macrovascular space. These findings highlight the extensive contribution to rs-fMRI blood-oxygenation level-dependent (BOLD) and FC predominantly by large veins, but also by large arteries. These findings pave the way for future studies aimed at more comprehensively modeling and thereby removing these macrovascular contributions.

## 1 Introduction

Resting-state blood-oxygenation level-dependent (BOLD) functional magnetic resonance imaging (rs-fMRI) is extensively used for mapping resting-state functional connectivity (rs-fcMRI) and for providing brain-health assessments (Srivastava et al., 2022;van den Heuvel & Hulshoff Pol, 2010). This technique has been used in aging, Alzheimer’s disease (Vemuri et al., 2012), and stroke (Ovadia-Caro et al., 2014), among others, and is commonly applied in studying brain development and aging processes (Andrews-Hanna et al., 2007;Geerligs et al., 2015). Most commonly, rs-fcMRI is calculated by correlation analysis (Bandettini et al., 1993;Biswal et al., 1995), in which the BOLD signals from seed voxels are correlated with those in the rest of the brain. High correlation is commonly interpreted as synchronous neural activity. It is possible, however, for large blood vessels in the brain to exhibit strong correlations among themselves as well as the global mean BOLD signal (Tong, Yao, et al., 2019). As the macrovascular system is responsible for supplying and draining blood from a large area of the brain, it is unlikely to be associated with local specific neural activity (Uludağ & Blinder, 2018), but rather with physiological noise and systemic processes. Thus, functional connectivity (FC) estimates may be biased by these macrovascular correlations (Huck et al., 2023;Kalcher et al., 2015). In order to understand macrovascular bias on FC estimates using rs-fMRI, it is imperative to understand the nature of signals that they carry, and the magnitude of their resultant BOLD signals.

The debate of macro- versus microvascular BOLD contributions in task-based fMRI has a long history. At a very early age, with both rodent experiments and simulation studies, Boxerman and colleagues demonstrated that the macrovasculature (defined as major cerebral sinuses and arteries) affects gradient-echo (GRE) BOLD contrast more than the microvasculature (defined as the capillary network, arterioles, and venules) (Boxerman et al., 1995). Although the sensitivity of microvasculature increases with an increase in the main magnetic field (Menon & Goodyear, 1999), veins still account for the majority of the signal fluctuation in BOLD signals at 7 T (Menon, 2012). In previous studies on rats, it was demonstrated that fcMRI could be used to detect venous structure (Hyde & Li, 2014) and at 11.7 T, the venous BOLD response is twice as high as that in brain tissue (Yu et al., 2012). Macrovascular BOLD, especially that in large veins, is defined as BOLD signal arising from veins with diameters exceeding 25 μm (Menon, 2002). Furthermore, multiple factors (for example, orientation and cortical depth of the blood vessel) are known to modulate macrovascular BOLD (Viessmann et al., 2019). It is also possible that factors such as spatial resolution and participant positioning may have an effect on the macrovascular effect. It is unclear how these factors interact with one another.

Unlike in conventional task-based fMRI, rs-fMRI focuses on the dynamic BOLD signal in the low-frequency band (Tong, Hocke, et al., 2019). Correspondingly, FC is computed largely from low-frequency BOLD fluctuations (Biswal et al., 1995;Josephs & Henson, 1999). Within this frequency range, a large body of work has demonstrated consistent time-lagged spatial correlational structures stemming from what has been termed systemic low-frequency oscillations (sLFOs) (Tong et al., 2015). These sLFOs permeate the brain volume, being particularly pronounced in the vasculature. The most commonly referenced contributors to the sLFOs would be vasomotion (Hundley et al., 1988;Mayhew et al., 1996;Rivadulla et al., 2011), heart rate variability (Thayer et al., 2012), respiratory volume variability (Birn et al., 2006;Chang et al., 2009), gastric oscillations (Mohamed Yacin et al., 2011;Rebollo et al., 2018), and variations in carbon dioxide levels (Golestani et al., 2015;Sassaroli et al., 2012;Wise et al., 2004). Notably, robust anti-correlations between arterial and venous BOLD signals in the low-frequency range have also been reported (Tong, Yao, et al., 2019), overturning the long-held belief that arterial BOLD implications are negligible, despite the diminutive arterial contrast.

Some may argue that it would be simple to identify and mask out regions with macrovasculature during rs-fcMRI analysis. However, according to the biophysical model of BOLD signal, the T_2_* relaxation effect caused by the susceptibility differences between blood and brain tissue originating from macrovasculature extends well beyond the voxel (Ogawa, Menon, et al., 1993), as recently shown in-vivo at 7 T (Huck et al., 2023). Moreover, previous results indicated that macrovascular signals and global BOLD signals are highly synchronous (Tong et al., 2015), suggesting that macrovasculature may likely contribute significantly to the gray matter (GM) BOLD signal. The potential spatial extent of macrovascular contribution to rs-fcMRI metrics in the perivascular tissue elevates the potential influence of macrovascular BOLD on rs-fcMRI and the challenge in removing it, and yet, it is not well understood.

This study was inspired by previous observations of robust correlations between arterial and venous signals in the low-frequency range (Tong et al., 2015), and by recent work extending the investigation into the rest of the venous vasculature at 7 T (Huck et al., 2023). Although these two previous studies are informative, they either only examine the correlation between a very limited number of large blood vessels (Tong et al., 2015) or only examine venous but not arterial vasculature (Huck et al., 2023). In addition, the macrovascular effect present in the perivascular tissue, despite its importance, has largely been overlooked, and existing work focuses on higher-order empirical model fitting, which may not fully capture the macrovascular contribution to perivascular tissue. The purpose of this study was to demonstrate macrovascular effects in both the macrovasculature and the perivascular tissue by utilizing time-of-flight imaging (TOF) at 3 Tesla to locate macrovasculature.

## 2 Methods

### 2.1 Participants

This study used data from the Midnight Scan Club (MSC) dataset, which comprises MRI data from 10 young, healthy, right-handed participants, five males and five females, ages 24–34 (Gordon et al., 2017). The study protocol was approved by the Human Studies Committee and Institute Review Board at Washington University School of Medicine in accordance with the Declaration of Helsinki. This data can be obtained from the OpenNeuro database, with accession number ds000224. The study was approved by Baycrest REB (#11-47).

### 2.2 MRI acquisition

Subjects underwent 12 imaging sessions on a Siemens TRIO 3 T MRI scanner (Siemens Healthcare GmbH, Erlangen, Germany) on separate days. In this section, only the protocols related to this study are listed.

In total, four T1-weighted data sets (sagittal, 224 slices, 0.8 mm isotropic resolution, TE = 3.74 ms, TR = 2400 ms, TI = 1000 ms, flip angle = 8^o^), 12 time-of-flight (TOF) 2D angiograms were acquired, including 4 transverse (“angiograms”, 0.6 x 0.6 x 1.0 mm, 44 slices, TR = 25 ms, TE = 3.34 ms), four sagittal (“venograms”, 0.8 x 0.8 x 2.0 mm thickness, 120 slices, TR = 27 ms, TE = 7.05 ms), and four coronal encoded (“venograms”, 0.7 x 0.7 x 2.5 mm thickness, 128 slices, TR = 28 ms, TE = 7.18 ms), were included for each participant. The participants were instructed to fixate on a white crosshair on a black background during the rs-fMRI scans (TR = 2.2 s, TE = 27 ms,α= 90^o^, 4 mm isotropic resolution, 36 slices, scan time = 30 min). An EyeLink 1000 eye-tracking system (SR-Research, Ottawa, Canada,http://www.sr-research.com) was used to ensure participant alertness during the scans.

### 2.3 Blood vessel segmentation and data processing

The strategies for macrovasculature segmentation and processing are summarized inFigure 1a. TOF data were registered to T1 space within each subject (FSL MCFLIRT, dof = 6, cost = corratio) and segmented using the Brain Charter Toolbox (Bernier et al., 2018). Visual inspection was performed to ensure the absence of artifacts. Vascular segmentations from all TOF data across all encoding directions were summed and then binarized to produce the final vascular segmentation. This was in turn downsampled to the rs-fMRI resolution. Due to the fact that all TOF data contained both arteries and veins, each arterial and venous map was manually separated after downsampling into different maps based on anatomical atlas (Tortora & Derrickson, 2018).

**Fig. 1.**
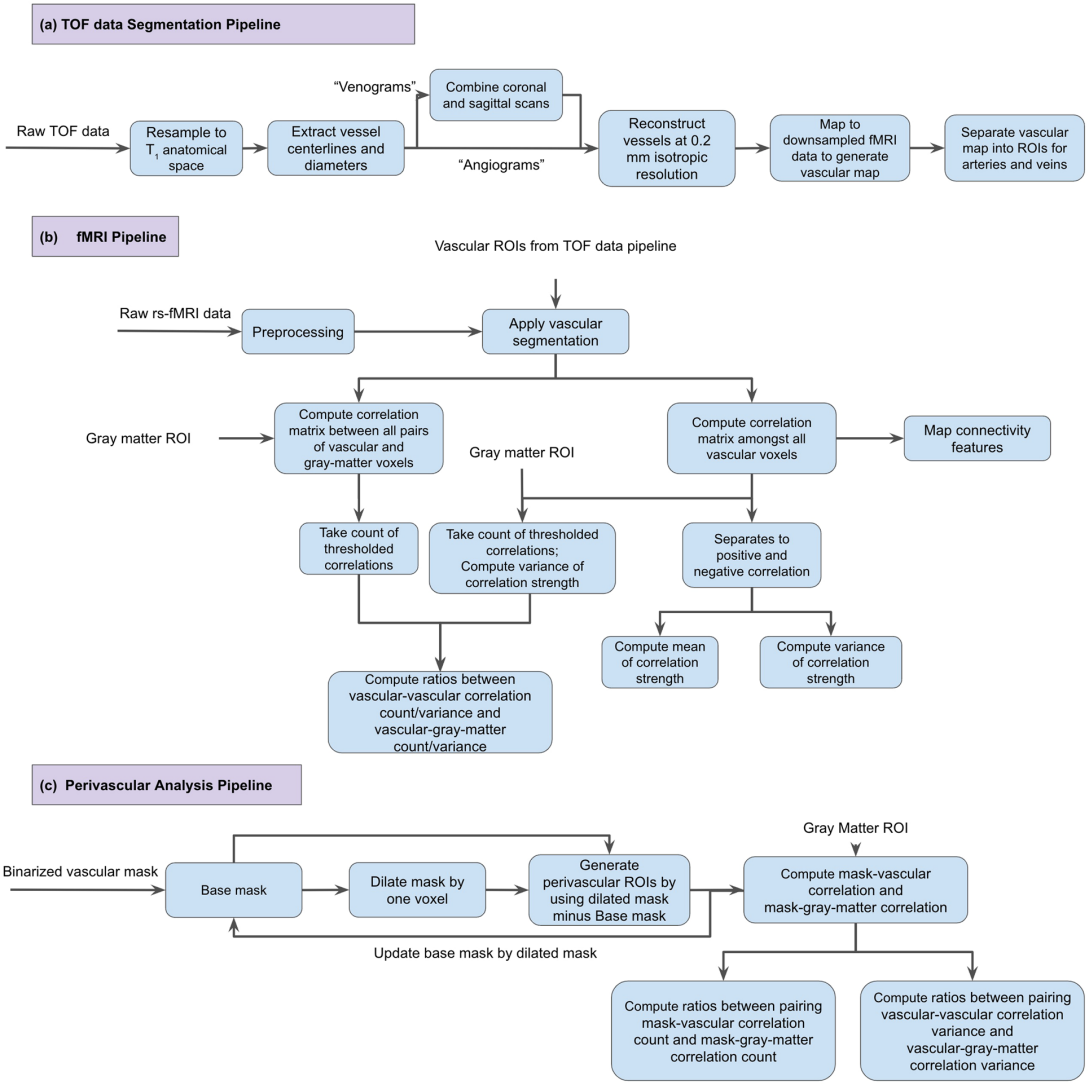
Overview of the analysis procedure. (a) The TOF data preprocessing and segmentation pipeline; (b) the fMRI analysis pipeline; and (c) the analysis pipeline for assessing the perivascular effects.

### 2.4 fMRI processing and FC analysis

A summary of the rs-fMRI processing procedures can be found inFigure 1b. fMRI preprocessing pipeline was implemented with tools from FSL (Jenkinson et al., 2012), AFNI (Cox, 1996), and FreeSurfer (Fischl, 2012). The following steps were included in the preprocessing steps: (a) 3D motion correction (FSL MCFLIRT), (b) slice-timing correction (FSL slicetimer), (c) brain extraction (FSL bet2 and FreeSurfer mri_watershed), (d) rigid body coregistration of functional data to the individual T1 image (FSL FLIRT), (e) regression of the mean signals from white-matter (WM) and cerebrospinal fluid (CSF) regions (fsl_glm), (f) bandpass filtering to obtain frequency band 0.01–0.1 Hz (AFNI 3dBandpass), and (g) the data were spatially smoothed with 6 mm full-width half-maximum (FWHM) Gaussian kernel (FSL fslmaths).

**Fig. 2.**
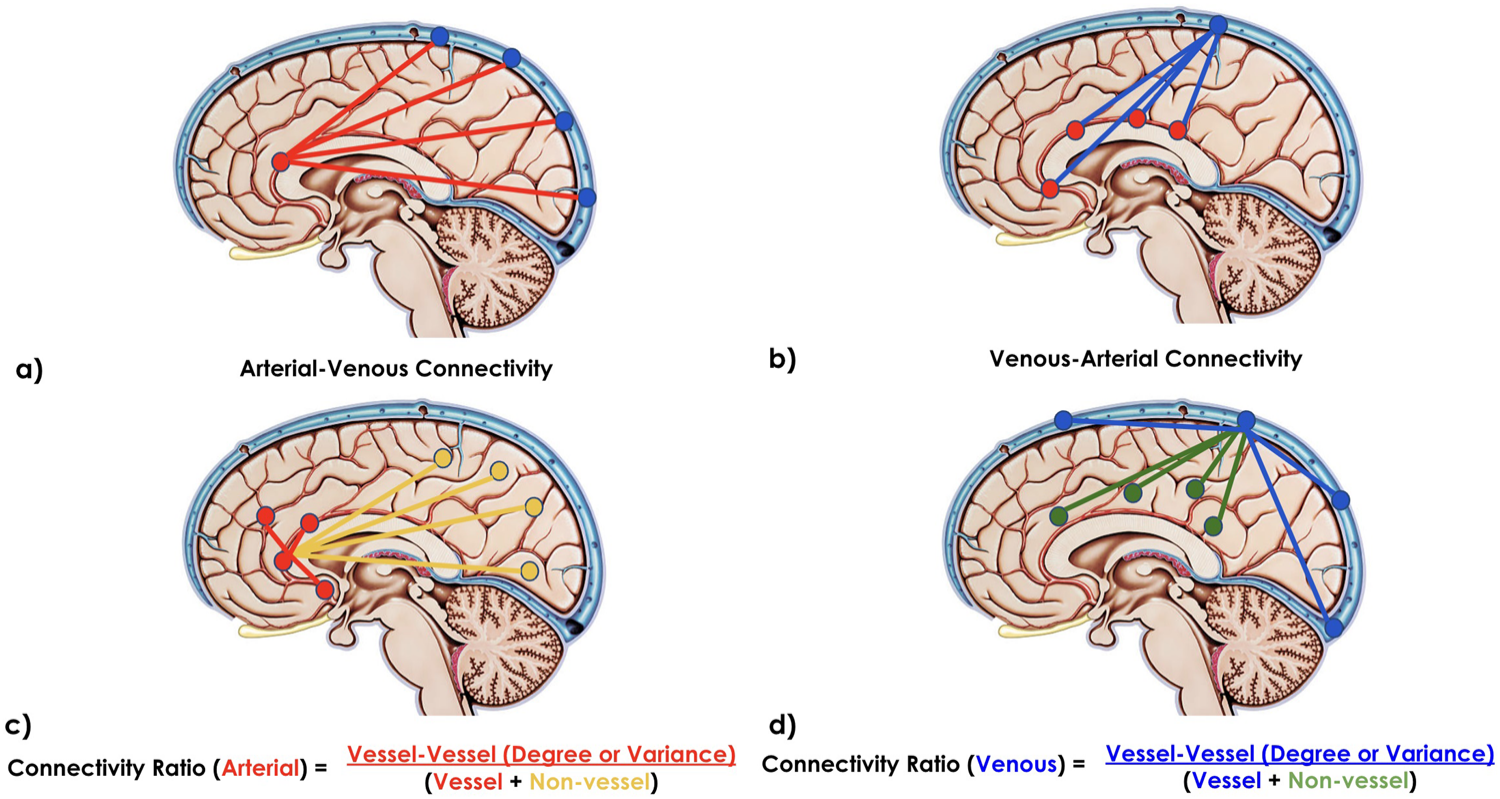
Illustration of measurement metrics (vascular cartoons are adapted fromJFalcetti, 2014). (a) Arterial-venous connectivity: each arterial seed voxel is connected to the entire venous network; (b) venous-arterial connectivity: each venous seed voxel is connected to the entire arterial network; (c) arterial-arterial connectivity ratio: the mean metrics associated with arterial-arterial connectivity is divided by the metrics of arterial-GM connectivity; and (d) venous-venous connectivity ratio: the mean metrics associated with venous-venous connectivity is divided by the metrics of venous-GM connectivity.

For each session of each participant, the voxel-wise vascular-driven connectivity metrics were calculated for each pair of voxels, namely (1) venous-venous correlation, defined as a venous seed voxel correlated with all other venous voxels; (2) arterial-arterial correlation, defined as an arterial seed voxel correlated with all other arterial voxels; (3) arterial-venous correlation, defined as an arterial seed voxel correlated with all venous voxels (and transposed to obtained venous-arterial correlation); and (4) venous-arterial correlation, defined as a venous seed voxel correlated with all arterial voxels ( Fig. 2 ). Then, inspired by previous research ( Buckner et al., 2009 ; Cole et al., 2010 ), we also computed the degree of connectivity (D) and strength of connectivity (S) as global metrics of connectivity. At each vascular voxel, D was computed by counting the number of voxels connected to each other vascular voxel and having a correlation coefficient greater than 0.15 ( Buckner et al., 2009 ; Cole et al., 2010 ). Voxel-wise connectivity strength (S) and spatial variance (σ ^2^ ) are computed as the mean and variance of correlations. That is, the spatial average and variance across all voxels correlating with each seed voxel (e.g., all arterial voxels in arterial-arterial correlations) are quantified. The maps of D, S, and σ ^2^ maps were further separated into:

- D_V,V_, S_V,V_, σ^2^_V,V_: based solely on venous-venous correlations;
- D_A,V_, S_A,V_, σ^2^_A,V_: based solely on arterial-venous correlations (arterial-venous correlation, computed within the arterial vasculature); (Fig. 2a)
- D_V,A_, S_V,A_, σ^2^_V,A_: based solely on venous-arterial correlations (venous-arterial correlation, computed within the venous vasculature); (Fig. 2b)
- D_A,A_, S_A,A_, σ^2^_A,A_: based solely on arterial-arterial correlation.

Moreover, correlation-strength maps are separated by positive and negative correlations.

### 2.5 Macrovascular contribution to rs-fcMRI

For a better understanding of the macrovascular contribution to overall brain connectivity, we also computed GM degree of connectivity, defined as the number of correlations between each GM voxel and each other GM voxel. These correlations were thresholded at 0.15, as mentioned earlier. When mapping the ratios of macrovascular to whole-GM degrees of connectivity, vascular masks were masked by GM masks to restrict the vasculature to those within the GM. Additionally, variance ratios were calculated by substituting variance of connectivity for degree of connectivity. These ratios were further separated into:

- Arterial-arterial, in terms of D_A,A_:D_A,GM_, σ^2^_A,A_:σ^2^_A,GM_; (Fig. 2c)
- Arterial versus venous, in terms of D_A,V_:D_A,GM_, σ^2^_A,V_:σ^2^_A,GM_, D_V,A_:D_V,GM_, σ^2^_V,A_:σ^2^_V,GM_;
- Venous-venous, in terms of D_V,V_:D_V,GM_, σ^2^_V,V_:σ^2^_V,GM_. (Fig. 2d)

#### 2.5.1 Perivascular effects on rs-fcMRI metrics

Figure 1cillustrates the methods used to assess the perivascular contributions by the macrovasculature. Briefly, we expanded the analysis described under the section*Macrovascular contributions to rs-fcMRI*into voxels next to those containing the macrovasculature. That is, we computed voxel-wise pairwise correlations stemming from perivascular voxels for arterial and venous macrovascular voxels (similar to the case of arterial and venous correlations described earlier), and then derived degrees of correlation from these perivascular voxels. The perivascular ROIs were generated as follows. First, the arterial and venous segmentations were dilated by 1 voxel in 3D. Second, the original vascular ROI was subtracted from the dilated vascular ROI. This produced the perivascular ROI that is 1 voxel distant from the voxel containing the vessel. This process was repeated for perivascular distances of 2 and 3 voxels. With the same approach as that used for the case of arterial and venous correlations, we computed ratios of macrovascular to whole-GM degrees (D_A(ex),A_:D_A(ex),GM_for the ratio between arterial perivascular tissue to arterial correlation degree and arterial perivascular tissue to GM correlation degree and D_V(ex),V_:D_V(ex),GM_for the ratio between venous perivascular tissue to venous correlation degree and venous perivascular tissue to GM correlation degree). Additionally, computed ratios of macrovascular to whole-GM variances (σ^2^_A(ex),A_:σ^2^_A(ex),GM_, for the ratio between arterial perivascular tissue to arterial correlation variance and arterial perivascular tissue to GM correlation variance and σ^2^_V(ex),V_:σ^2^_V(ex),GM_for the ratio between venous perivascular tissue to venous correlation variance and venous perivascular tissue to GM correlation variance) were arrived at by substituting σ^2^for the D in the computation of degree ratio maps mentioned previously.

### 2.6 Macrovascular fraction in typical resting-state networks

For reference, we computed the mean likelihood of observing large vessels in a set of RSNs derived from the Human Connectome Project (Nozais et al., 2023), based on previously published macrovascular frequency maps (Viviani, 2016) (Table 1). The RSNs were thresholded by z-value higher than 3, and macrovascular frequency maps were thresholded by 90 percentile of signal intensity. The ratio was calculated based on the percentage of RSNs occupied by macrovasculature.

**Table 1.** List of percent macrovascular voxel occupancy well-established RSNs.

RSN name	Percentage of RSN occupied by macrovasculature (%)
Anterior cingulate	8.05
Med. Post. Occ. / Primary visual	6.93
Insula / Salience	6.55
Cingulo-opercular	5.97
Primary auditory	5.2
Posterior Occipital	4.63
Medial Occipital	3.13
PH-Precuneal / Parahippocampal DMN	3.09
Inf. Central / SM head	2.66
Posterior cingulate-Precuneal / Dorsal DMN	2.32
Precuneus / DMN proper	2.04
Precentral / Motor	1.68
Medial-frontal	1.52
Left Inferior frontal / Language production	1.42
Dorsolateral prefrontal cortex	1.32
Superior Parietal / Dorsal attention	1.23
Mid. Central (R) / SM hand (L)	1.00
Mid. Central (L) / SM hand (R)	0.89
FPT 1 (L)	0.87
PP-Precuneal / Posterior DMN	0.8
Anterior Superior Parietal	0.73
Superior Central / sensorimotor body	0.68
FPT 1 (R)	0.5
FPT 3 (R)	0.5
Posterior Superior Parietal	0.36
FPT 2 (L)	0.33
Lateral Posterior Occipital	0.21
Lateral Occipital	0.14
FPT 3 (L) / Language comprehension	0.14
FPT 2 (R)	0.13

This table summarizes the percentage of voxels occupied by macrovasculature for each RSN. A high percentage (above 5%) of occupancy was observed in primary visual, primary auditory, anterior cingulate, salience, and cingulo-opercular areas. DMN = default-mode network. FPT = Fronto-parieto-temporal network, L = left, R = right.

## 3 Results

A sample vascular segmentation is shown inFigure 3.

**Fig. 3.**
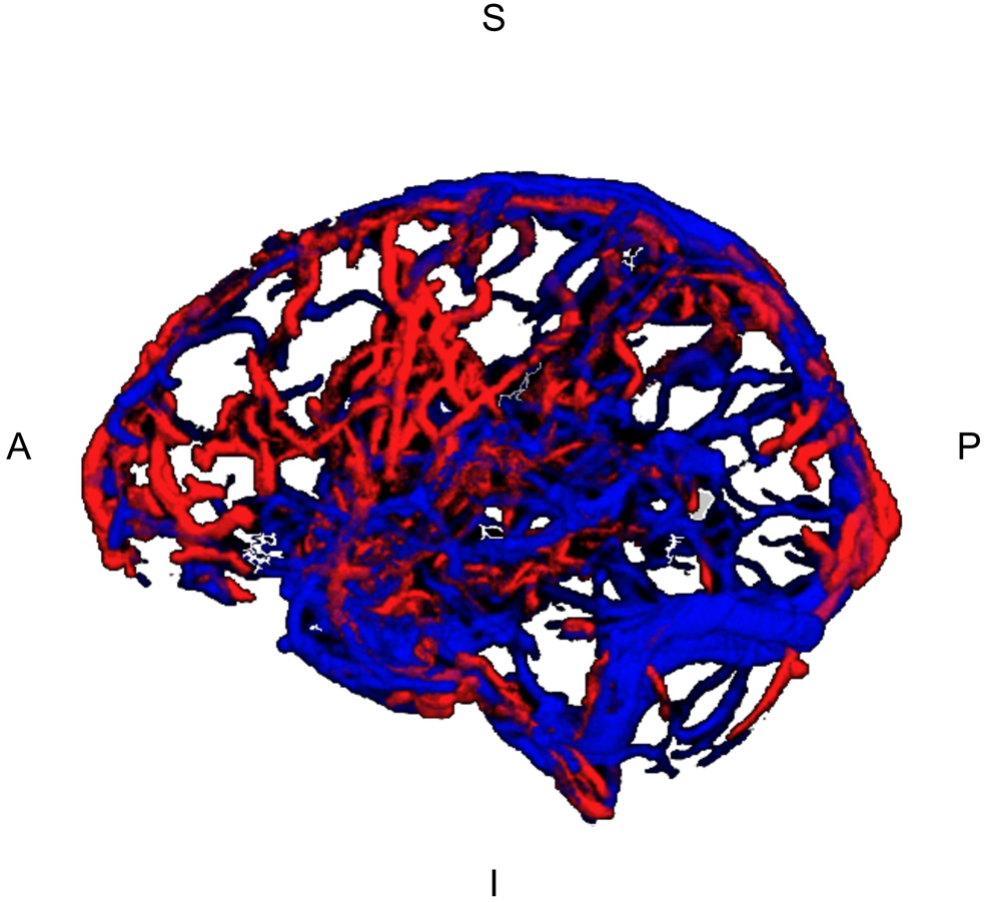
Sample segmented blood vessels (Before separating the venous and arterial manually). Red: segmented based on MRAs; blue: segmented based on MRVs. I = inferior, S = superior, A = anterior, P = posterior.

### 3.1 Macrovascular connectivity

Moreover, the key findings are summarized inFigure 4. In order to assess the connectivity arising from macrovasculature, three metrics were used: degree of connectivity (D) (Fig. 5), strength of connectivity (S) (Fig. 6), and variance of connectivity (σ^2^) (Fig. 7).

**Fig. 4.**
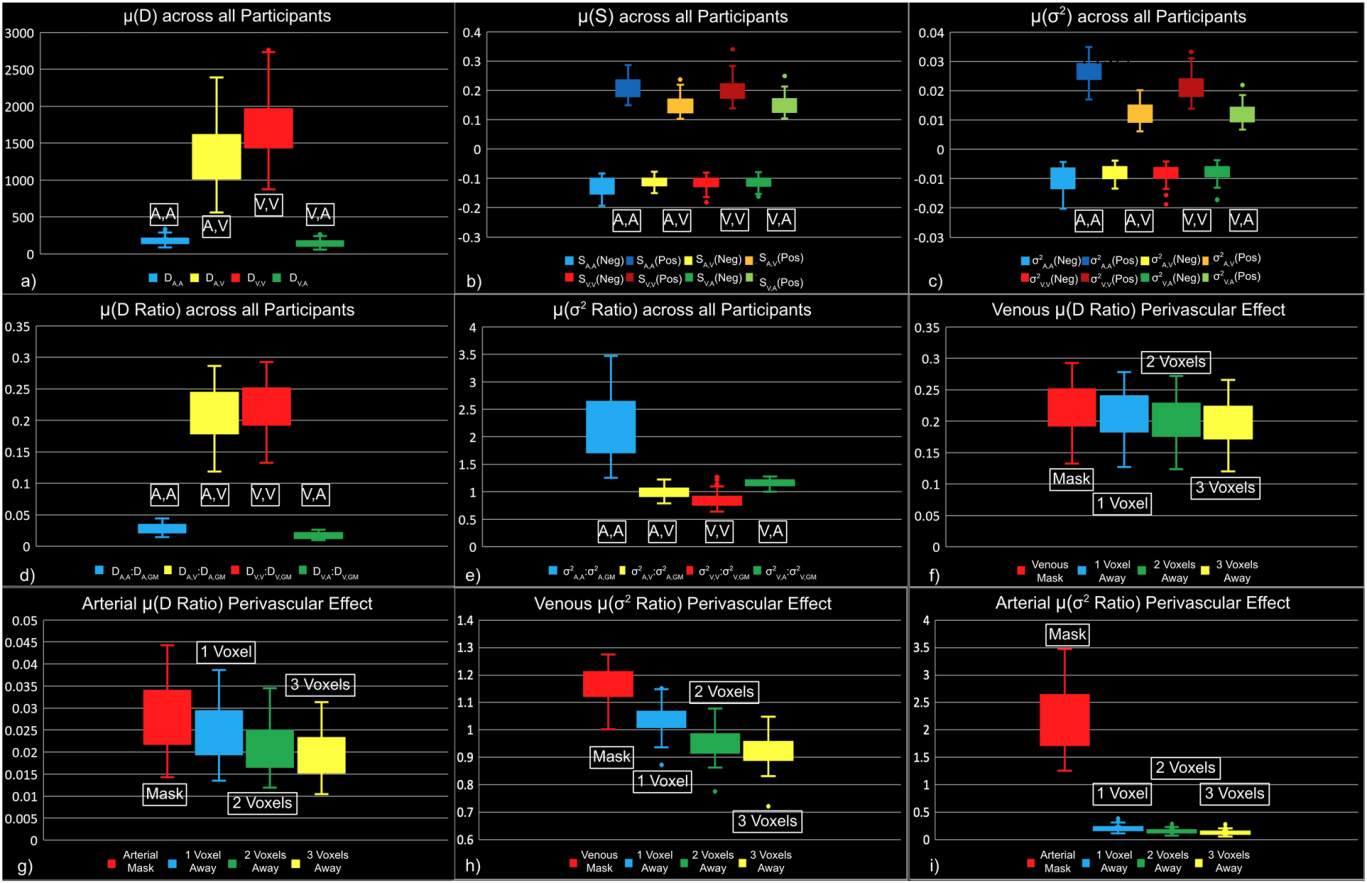
Summary box plots for each metric, with the same colour encoding asFigures 5–11. (a) Degree of connectivity; (b) mean strength of connectivity; (c) variance of connectivity; (d) ratio of degree of connectivity; (e) ratio of variance of connectivity; (f) venous perivascular ratio of degree of connectivity; (g) arterial perivascular ratio of degree of connectivity; (h) venous perivascular ratio of variance of connectivity; and (i) arterial perivascular ratio of variance of connectivity. Metrics are plotted for positive and negative correlations separately. The whiskers represent the extent of the group-wise first and fourth quartiles.

**Fig. 5.**
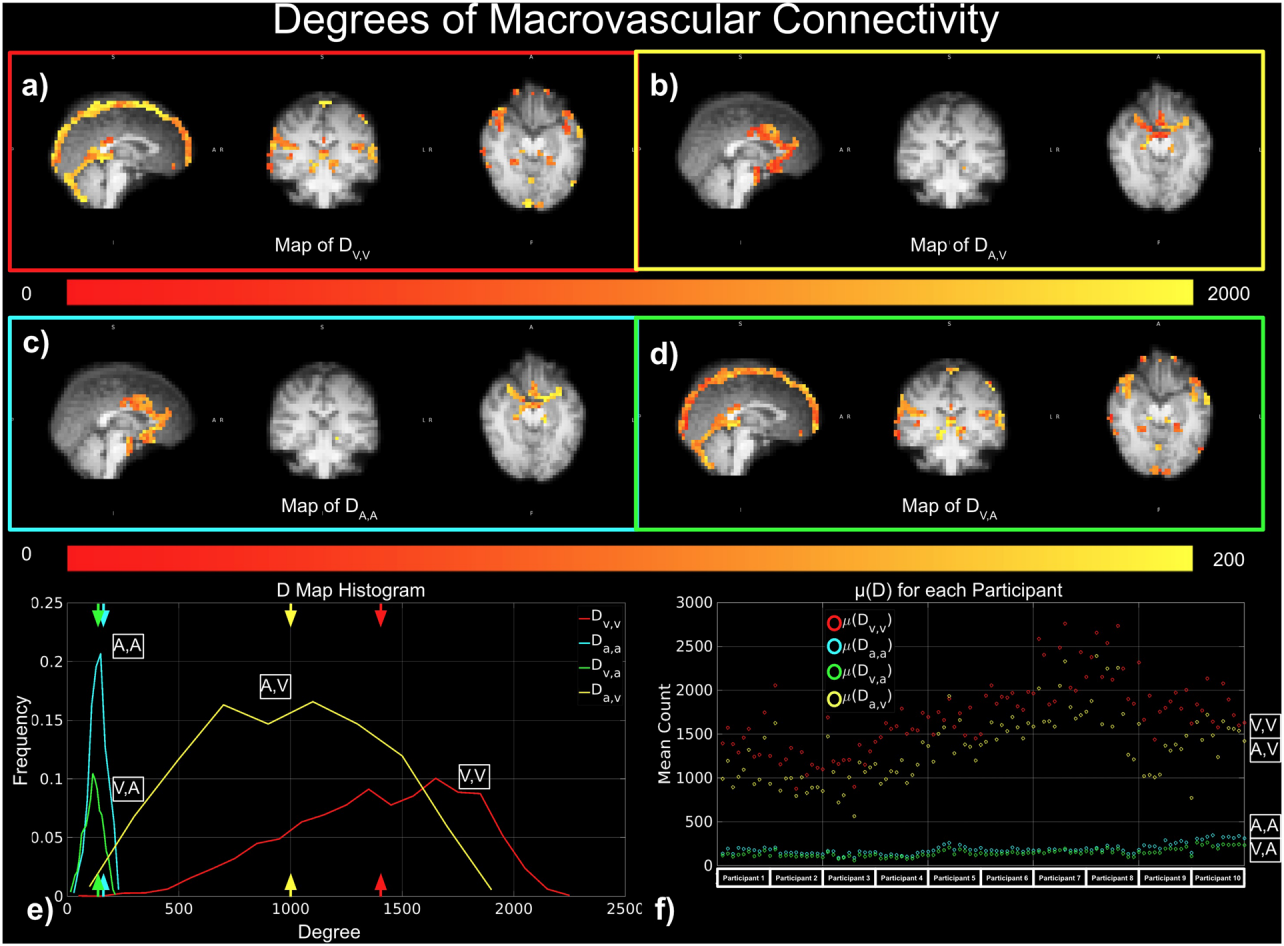
Degrees of macrovascular connectivity. Red indicates D_V,V_; yellow indicates D_A,V_; cyan indicates D_A,A_; and green indicates D_V,A_. (a)-(e) represent results from a representative data set, while (f) shows results from all data sets.

**Fig. 6.**
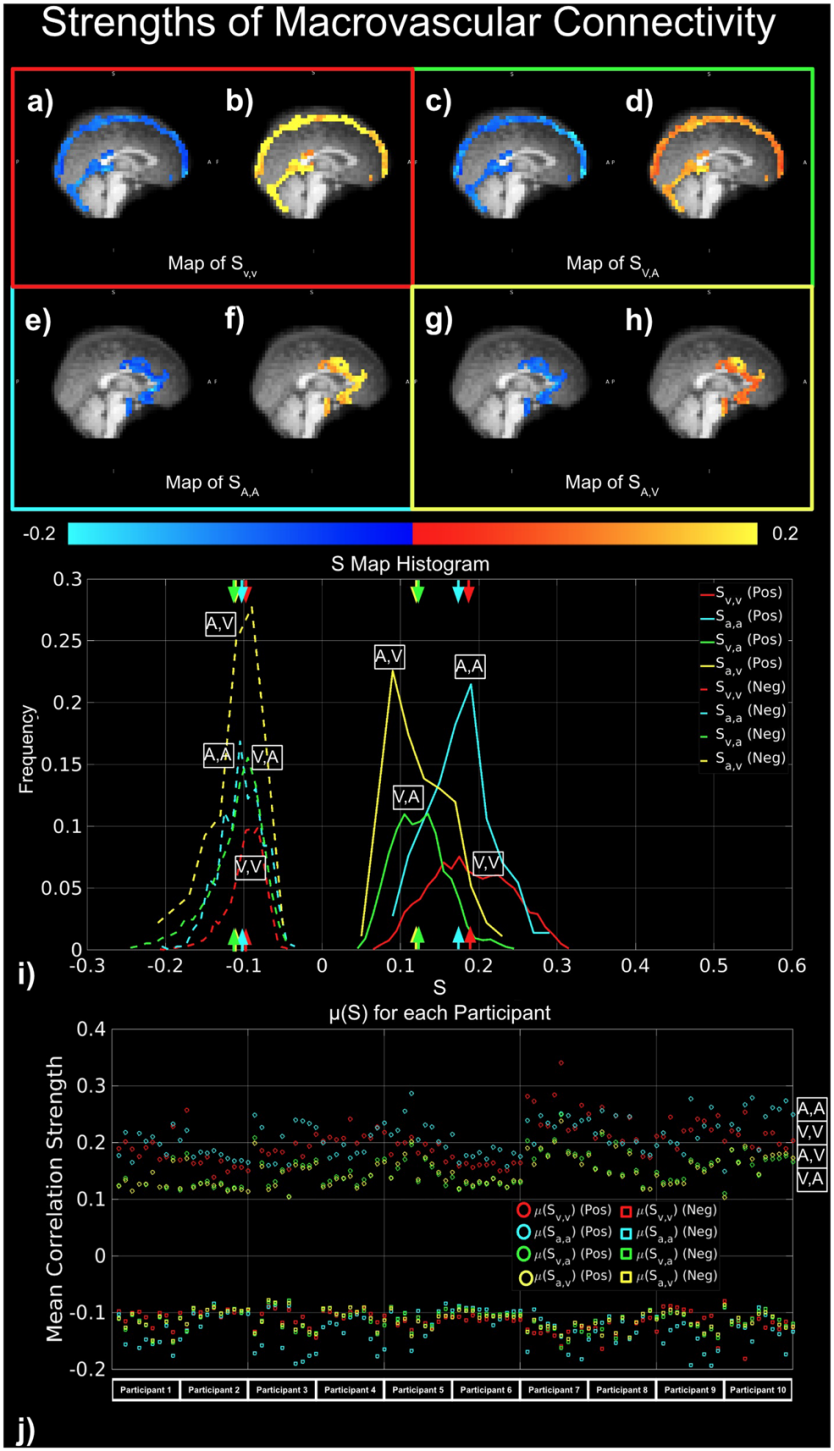
Strengths of macrovascular connectivity. Red indicates S_V,V_; yellow indicates S_A,V_; cyan indicates S_A,A_; and green indicates S_V,A_. (a)-(i) represent results from a representative data set, while (j) shows results from all data sets correlation with positive S represented by circles, and correlation with negative S represented by squares.

**Fig. 7.**
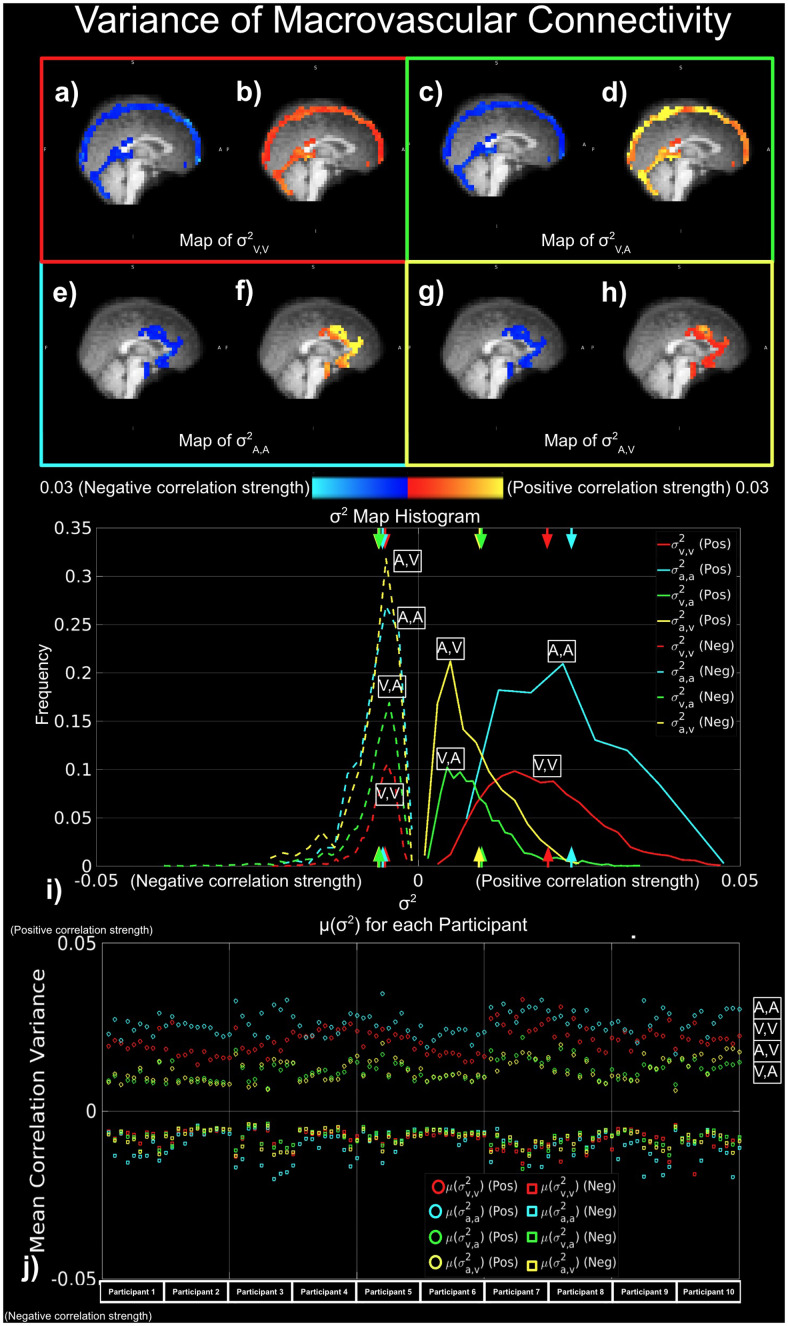
Variance of macrovascular connectivity. Red indicates σ^2^_v,v_; yellow indicates σ^2^_A,V_; cyan indicates σ^2^_A,A_; and green indicates σ^2^_V,A_, with correlation with positive S represented by circles, and correlation with negative S represented by squares. (a)-(i) represent results from a representative data set, while (j) shows results from all data sets.

#### 3.1.1 Degree of macrovascular connectivity

InFigure 5, we demonstrated strong apparent FC within the macrovascular regions based on degree of connectivity. As seen in the results from a representative dataset, voxels with non-zero degrees of vascular-related connections (D_V,V_, D_A,A_, D_A,V_, and D_V,A_) were found to span the entire macrovascular ROI, with higher values at the site of larger vessels (e.g., the superior sagittal sinus and Circle of Willis) (Fig. 5a-d). The histograms of D values show that D_V,V_and D_A,V_are higher than D_V,A_and D_A,A_, with the maximum degree exceeding 2000 (Fig. 5e). These patterns are also evident in μ(D) for these four correlation categories across all participants in all scan sessions (Fig. 5f).

There was also evidence of strong apparent connectivity within macrovasculature based on the strengths and variances metrics (Figs. 4,6and7). As seen in the summary results inFigure 4band in the representative dataset as used in the previous figures, higher S_V,V_magnitudes are more associated with positive than negative correlations (Fig. 6a, b). Likewise, higher S_A,A_magnitudes are also more associated with positive than negative correlations (Fig. 6e, f). Conversely, S_V,A_was similar in magnitude for both negative and positive correlations (Fig. 6c, d,g, h). These differences are further illustrated by the whole-vasculature histograms, with S_A,A_and S_V,V_showing higher magnitudes than S_A,V_and S_V,A_(Fig. 6i). Moreover, as shown across all subjects (Fig. 6j), the strong S_A,A_and S_V,V_patterns are observed across all participants in all scan sessions, outweighting values for S_A,V_and S_V,A_.

Similar to the mean correlation strength S, the correlation variance σ^2^_A,A_and σ^2^_V,V_are higher for positive than negative correlations, as shown for the same representative data set as in previous figures (Fig. 7a, b,e, f). This is confirmed in the group results inFigure 4c. There was no apparent difference in σ^2^_A,V_and σ^2^_V,A_between positive and negative correlations (Fig. 7c, d,g, h). Moreover, the frequency histograms showed the same patterns as maps, which suggest (Fig. 7i), a pattern that is consistent across all sessions from all participants exhibited the same pattern (Fig. 7j).

### 3.2 Contribution of the macrovascular connectivity to GM functional connectivity

**Fig. 8.**
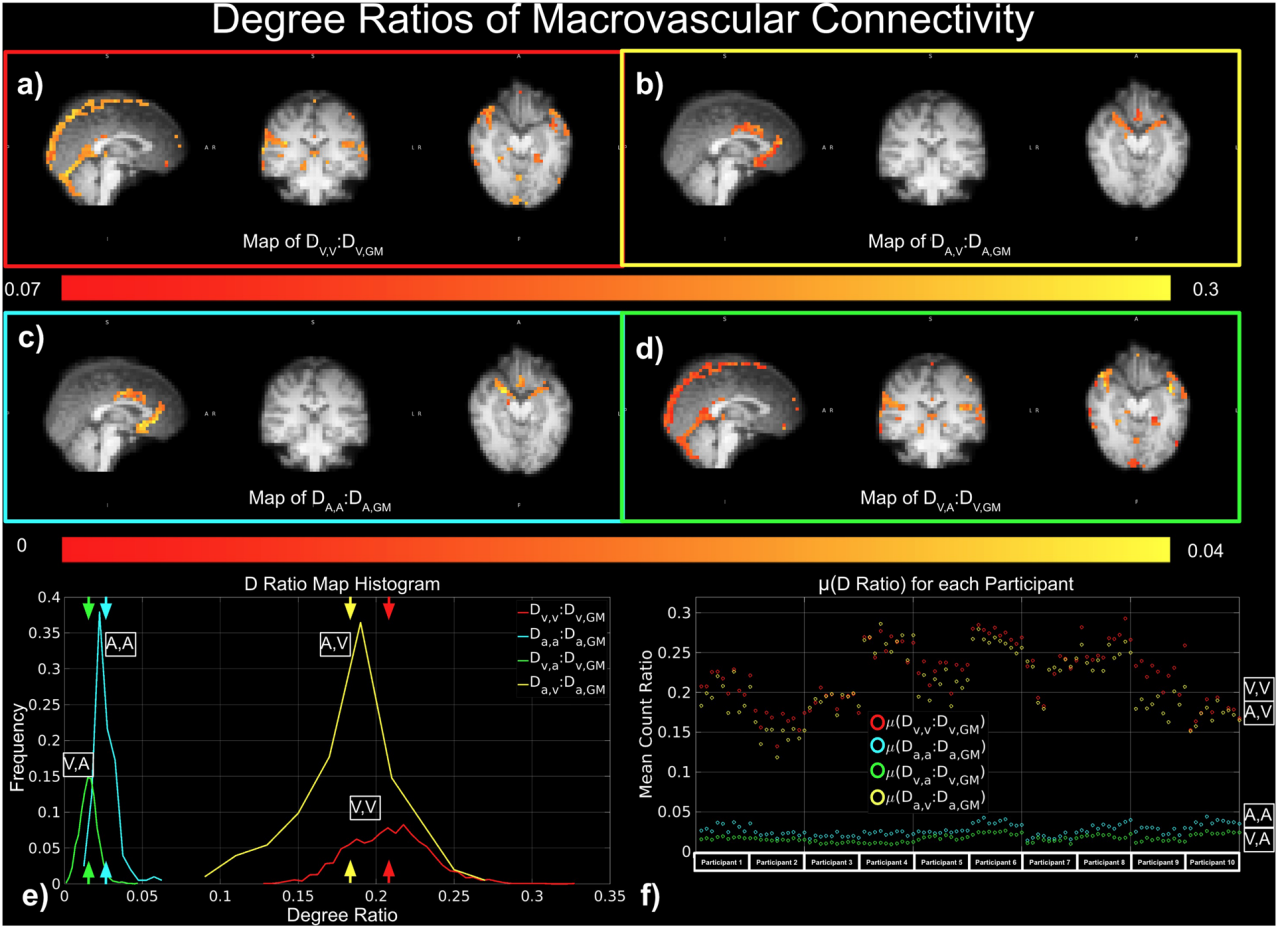
Contribution of the macrovascular connectivity to overall tissue degree of connectivity. The contribution was quantified as ratios between vascular and tissue degrees of connectivity, for example, D_v,v_:D_v,GM_, D_v,a_:D_v,GM_, D_a,v_:D_a,GM_, and D_a,a_:D_a,GM_. (a)-(e) Represent results from a representative data set, while (f) shows results from all data sets. Red indicates D_v,v_:D_v,GM_; yellow indicates D_a,v_:D_a,GM_; cyan indicates D_a,a_:D_a,GM_; and green indicates D_v,a_:D_v,GM_.

As shown inFigure 4d, alarge portion of the significant correlation in D_GM_could come from the vascular ROIs. This is confirmed in individual ratios (D_V,V_:D_V,GM_, D_V,A_:D_v,GM_, D_A,V_:D_A,GM_, and D_A,A_:D_A,GM_) (Fig. 8). The arterial ratios D_A,A_:D_A,GM_(Fig. 8c) and D_V,A_:D_V,GM_(Fig. 8b) are lower in comparison (Fig. 4d). These differences are further illustrated by the histograms, which show the degree ratios to be highest for D_V,V_:D_V,GM_, followed by D_A,V_:D_A,GM_, D_A,A_:D_A,GM_, and the lowest for D_V,A_:D_V,GM_(Fig. 8e). These patterns were also evident in mean degree ratios for the above four groups of correlations across all participants in all scan sessions (Fig. 8f).

**Fig. 9.**
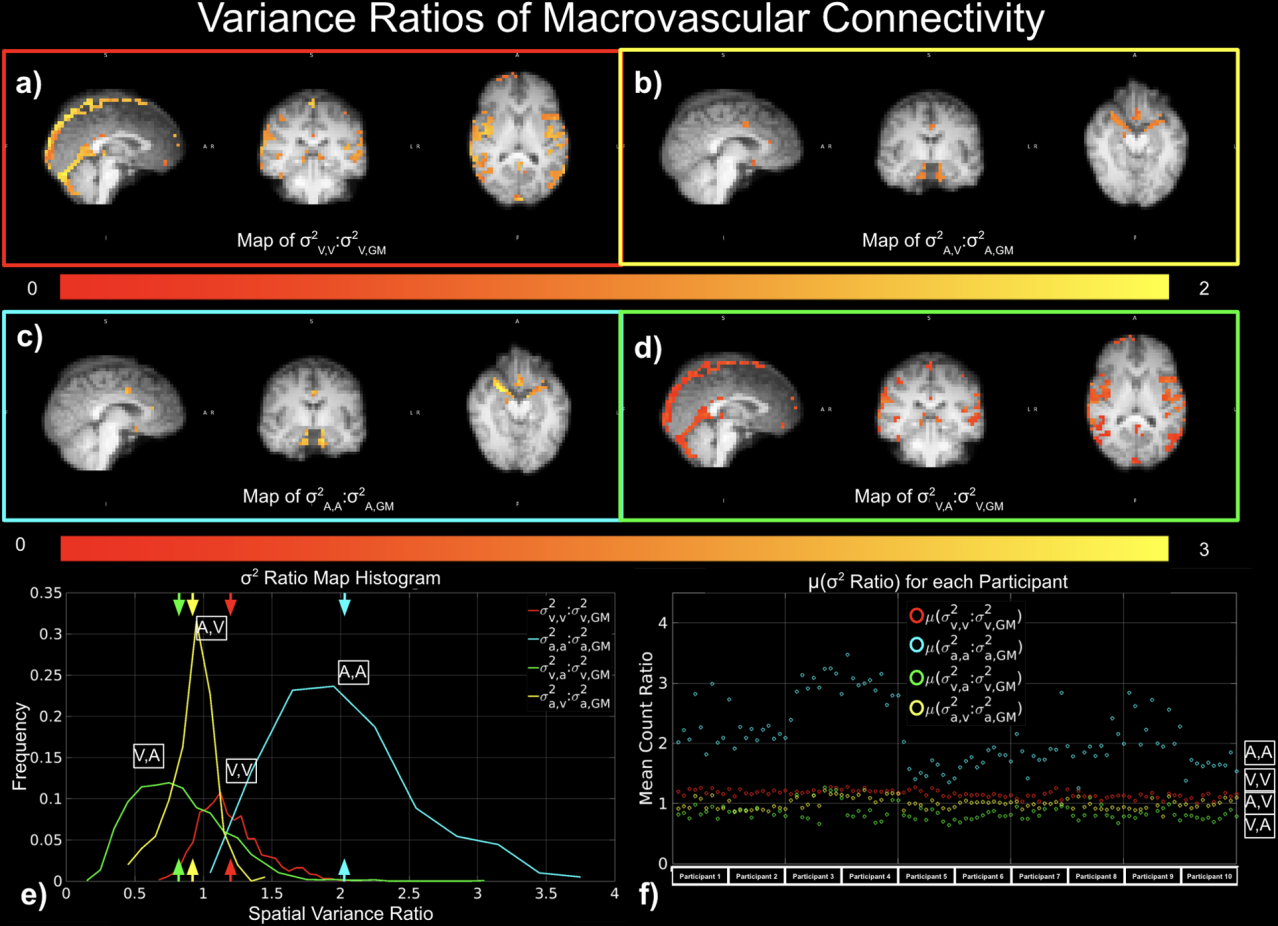
Contribution of the macrovascular connectivity to overall tissue variance of connectivity. The contribution was quantified as ratios between vascular and tissue variances of connectivity, for example, σ^2^_v,v_:σ^2^_v,GM_, σ^2^_v,a_:σ^2^_v,GM_, σ^2^_a,v_:σ^2^_a,GM_, and σ^2^_a,a_:σ^2^_a,GM_. Red indicates σ^2^_v,v_:σ^2^_v,GM_; yellow indicates σ^2^_a,v_:σ^2^_a,GM_; cyan indicates σ^2^_a,a_:σ^2^_a,GM_; and green indicates σ^2^_v,a_:σ^2^_v,GM_. (a)-(e) represent results from a representative data set, while (f) shows results from all data sets.

As a measure of the spatial variability of functional connectivity associated with the macrovasculature relative to that of whole GM, variance ratios (σ^2^_V,V_:σ^2^_V,GM_, σ^2^_V,A_:σ^2^_v,GM_, σ^2^_A,V_:σ^2^_A,GM_, and σ^2^_A,A_:σ^2^_A,GM_) between vascular and GM degrees of connectivity were computed. We found that the variance ratio σ^2^_A,A_:σ^2^_A,GM_(Fig. 9a) exhibited the highest values, followed by σ^2^_V,A_:σ^2^_v,GM_(Fig. 9b). σ^2^_V,V_:σ^2^_v,GM_(Fig. 9c) and σ^2^_A,V_:σ^2^_A,GM_(Fig. 9d). The group-average results are summarized inFigure 4. According to the histogram (Fig. 9e), the μ(σ^2^_V,V_:σ^2^_v,GM_) and μ(σ^2^_V,V_:σ^2^_v,GM_) had slightly different maxima, with μ(σ^2^_V,V_:σ^2^_v,GM_) higher than the μ(σ^2^_V,V_:σ^2^_v,GM_). Across all sessions for all participants, we observed that the μ(σ^2^_A,A_:σ^2^_A,GM_) was consistently higher than variance ratios for any connectivity involved veins (Fig. 9f).

### 3.3 Dependence of perivascular connectivity on the distance from vasculature

**Fig. 10.**
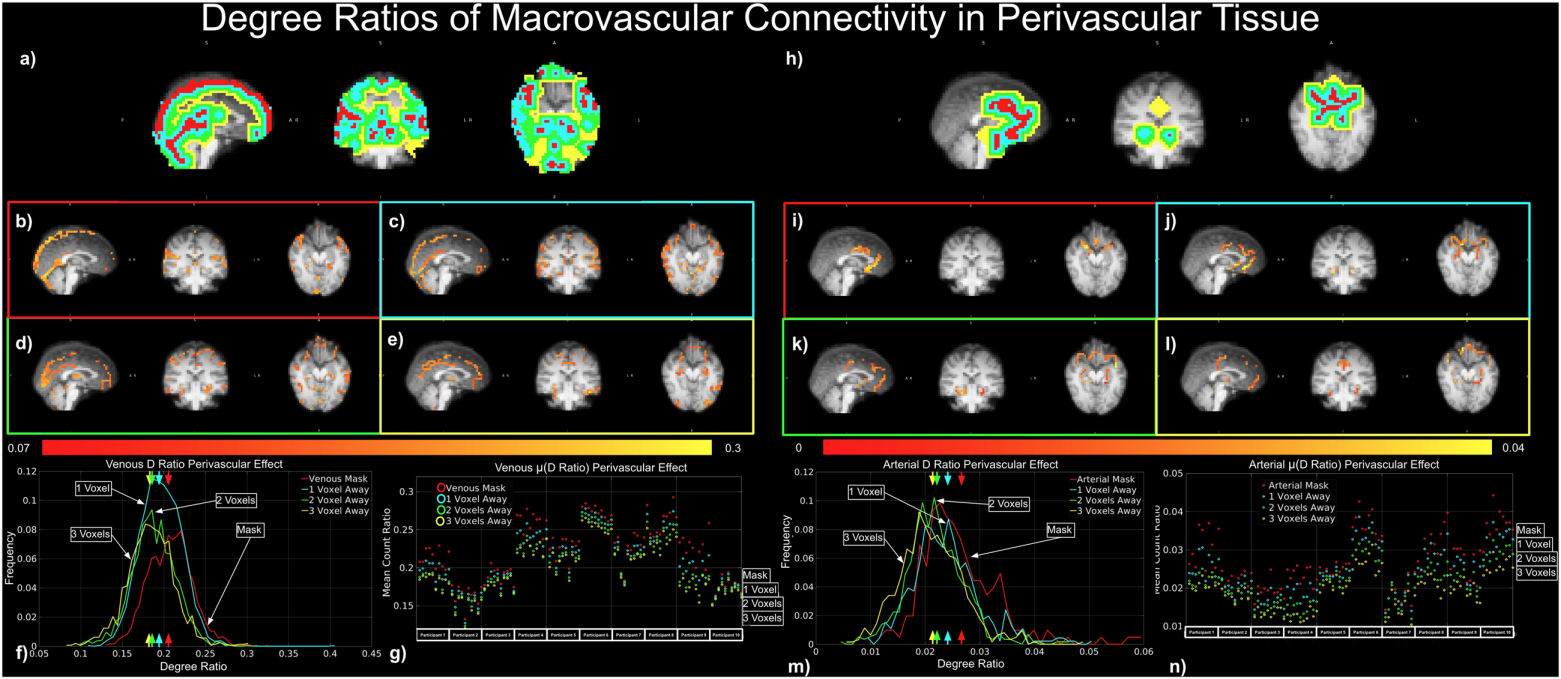
Dependence of perivascular degree of connectivity on distance from vasculature. The perivascular contribution of the macrovasculature to tissue connectivity is given by degree ratios D_v(ex),v_:D_v(ex),GM_(a-g) and D_a(ex),a_:D_a(ex),GM_(h-n). All plots represent results from the same representative data set, except for (g) and (n), which show results from all data sets. Perivascular ROIs with different distances to the macrovasculature are shown for both arteries and veins, with the color coding for perivascular distance defined as: red—voxel containing vasculature, blue—one voxel away, green—two voxels away, and yellow—three voxels away (a, h).

**Fig. 11.**
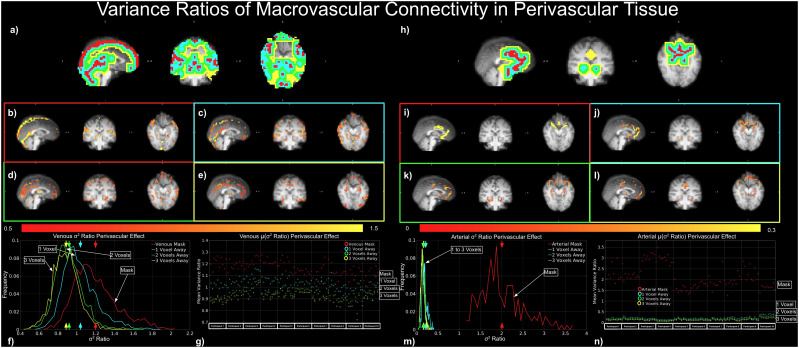
Dependence of perivascular variance of connectivity on distance from vasculature. An illustration of σ^2^_v(ex),v_:σ^2^_v(ex),GM_(a-g) and σ^2^_a(ex),s_:σ^2^_a(ex),GM_(h-n). Dilated vascular masks are shown for both arterial and venous, with color coding as: red—voxel containing vasculature, blue—one voxel away, green—two voxels away, and yellow—three voxels away (a, h). All plots represent results from the same representative data set, except for (g) and (n), which show results from all data sets.

The perivascular contribution of the macrovasculature to tissue connectivity is given by degree ratios (D_A(ex),A_:D_A(ex),GM_and D_A(ex),A_:D_A(ex),GM_) (Fig. 10) and variances ratios (σ^2^_V(ex),V_:σ^2^_V(ex),GM_and σ^2^_A(ex),A_:σ^2^_A(ex),GM_) (Fig. 11).

#### 3.3.1 Perivascular contribution to tissue connectivity: connectivity degree

The macrovascular contribution to perivascular tissue is summarized inFigure 4f, and as reflected by a representative subject (the same as used in the previous figures), D_V(ex),V_:D_V(ex),GM_decreases with increasing distance from the vasculature (Fig. 10b-e). A similar trend was observed for D_A(ex),A_:D_A(ex),GM_(Figs. 4gand10i-l). For the perivascular degree ratios D_V(ex),V_:D_V(ex),GM_and D_A(ex),A_:D_A(ex),GM_across the ROIs, the histogram demonstrated the same trend (Fig. 10f, m). As shown by the plots, the evidence above was consistent across all sessions and participants (Fig. 10g, n).

#### 3.3.2 Perivascular contribution of the macrovasculature to tissue connectivity: Connectivity variance

Similar perivascular contribution effects were also observed on the variance ratios (Fig. 4h, i). Based on the results of the same sample participant, both σ^2^_V(ex),V_:σ^2^_V(ex),GM_(Fig. 11b-e) and σ^2^_A(ex),A_:σ^2^_A(ex),GM_(Fig. 11i-l) decreased with increasing distance from the macrovascular system. It is also noteworthy that μ(σ^2^_V(ex),V_:σ^2^_V(ex),GM_) was higher than μ(σ^2^_A(ex),A_:σ^2^_A(ex),GM_) except for the macrovascular ROIs (Fig. 11f, m). Throughout all sessions and participants, the evidence above was consistent, and the variances were negligible after one voxel away from the large arteries (Fig. 11g, n).

## 4 Discussion

In rs-fMRI functional connectivity (FC) mapping, synchronized neural-related activation is assumed to be the major factor ( Biswal et al., 1995 ; Pan et al., 2015 ), and there has been little investigation of FC resulting from macrovascular signal fluctuations. In our previous study, a robust correlation was observed between the internal carotid artery and the internal jugular vein, as well as arteries and arteries ( Tong, Yao, et al., 2019 ). This work expands the previous study to include all detectable major veins as well as arteries in the brain in a systematic analysis of the macrovascular contribution to whole-GM functional connectivity. The main results are:

1. The macrovasculature consistently exhibited strong correlational connectivity among itself, with the sign of the correlations varying between arterial and venous connectivity;
2. GM connectivity was found to have a strong macrovascular contribution, stronger from veins than arteries;
3. FC originating from the macrovasculature displayed disproportionately high spatial variability compared to the spatial FC spatial variability across all GM voxels;
4. Macrovascular contributions to connectivity were still evident well beyond the confines of the macrovascular space.

### 4.1 The resting-state macrovascular BOLD effect

This is the first study to our knowledge that explores the contribution of the entire macrovasculature (to the best of our segmentation’s ability) to rs-fcMRI. In this study, we demonstrated that macrovascular contributions may bias rs-fMRI connectivity analysis, which may result in an incorrect interpretation of full brain connectivity and RSNs. An earlier review article proposed the concept of systematic low-frequency oscillations (sLFOs), which are defined as a vasogenic low-frequency BOLD signal traveling through the brain (Tong et al., 2015). It appears that the oscillation could be caused by vasomotion (Hundley et al., 1988;Mayhew et al., 1996;Rivadulla et al., 2011), heart-rate variability (Thayer et al., 2012), respiratory volume variability (Birn et al., 2006;Chang et al., 2009), gastric oscillations (Mohamed Yacin et al., 2011;Rebollo et al., 2018), and variations in carbon dioxide levels (Sassaroli et al., 2012;Wise et al., 2004), some of which originate outside the brain (Frederick & Tong, 2010;Li et al., 2018;Tong & Frederick, 2010). Furthermore, previous studies using near-infrared spectroscopy have shown that such oscillations travel through the vasculature, and that strong and vascular-specific spatial structures can be captured in the macrovasculature by regressing finger-tip oxygenation time courses with the whole-brain BOLD signal (Frederick et al., 2013;Tong & Frederick, 2012,^2014^;Tong, Bergethon, et al., 2011;Tong, Hocke, et al., 2011;Tong, Lindsey, et al., 2011;Tong et al., 2014,^2015^). Given the predominance of the BOLD signal by venous blood (Ogawa, Lee, et al., 1993;Segebarth et al., 1994), such sLFOs may indicate that some findings derived from rs-fMRI may be the result of venous bias rather than neural activity (Aso et al., 2020). Even if some of these mechanisms may have neuronal origins, the presence of this venous bias, which extends well beyond the vessels in GE-BOLD, leads to reduced BOLD spatial specificity.

### 4.2 Macrovascular connectivity

Our investigation of macrovascular connectivity is largely influenced by our previous work (Tong et al., 2015). In spite of the fact that their study only included a few very large arteries and veins, they were successful in showing a strong cross-correlation between large blood vessels, as well as between the large blood vessels and the global BOLD signal. Additionally, their results suggest that accounting for lags between time courses may be important for assessing macrovascular connectivity. Nevertheless, cross-correlation accounting for lags is only one of the possible methods used in FC analysis, which means that macrovascular effects observed using simple Pearson’s correlations may or may not be comparable. Moreover, it would be necessary to investigate whether the correlation can also be observed in the rest of the macrovasculature.

In line with the previous study discussed above, this study showed a strong correlation between BOLD fMRI signals from arterial and venous voxels (Figs. 5and6). As a point of clarification, our correlation results were based on Pearson’s correlation rather than cross-correlation, motivated by the stronger relevance of the former to functional connectivity mapping. Thus, unlike in our previous work (Tong, Yao, et al., 2019), arterial-venous time delays were not considered in calculating the correlation. Nonetheless, like that we found that the correlations between venous BOLD signals were mainly positive, whereas the correlations between arterial and venous BOLD signals were mainly negative. Thus, the results demonstrated in the IJV and ICA in the previous work are largely generalizable to other large vessels. A key difference between arteries and veins is their magnetic susceptibility, which is proportional to oxygenation and drives the amplitude of the BOLD signal (Ogawa, Menon, et al., 1993;Spees et al., 2001). At 3 Tesla, which is used in this study, the magnetic susceptibility of brain tissue and fully oxygenated blood is ~ -9.05 ppm while that of deoxygenated blood is ~ -7.9 ppm (Duyn & Schenck, 2017). Compared to the GM parenchyma, veins have a lower oxygenation level and contain paramagnetic blood, while large arteries have a higher oxygenation and contain diamagnetic blood. Thus, we expect the arterial-venous intravascular BOLD-signal oscillations to be naturally anti-correlated (not considering the interfering effect of temporal delay). These arterial-arterial and venous-venous anti-correlations were also observed by our previous study as shown by the histograms of the peak cross-correlation coefficients. However, in our results, not all arterial-arterial correlations were positive, and the same was true for venous-venous correlations. It is possible that the phenomenon is a result of the combination of intravascular and perivascular signals. As we have shown, in many cases, the intravascular and perivascular signals may be anti-correlated (Fig. 6), so it is conceivable that partial-voluming contributions from the two within the same voxel leads to a weighted sum of correlations from both sources.

In order to provide a better global view of the macrovascular contribution to FC, we additionally computed the degree of connectivity (D) and the strength of connectivity (S) as defined in (Buckner et al., 2009;Cole et al., 2010). Calculated as the number of voxels that have high correlation coefficients above the threshold, D represents the number of voxels that may be influenced by macrovascular signals. S, on the other hand, represents the mean strength of the macrovascular contribution. In our results, we found D_v,v_and S_v,v_higher than D_V,A_and S_V,A_(or D_A,V_and S_A,V_), which is the same as the previous results of superior sagittal sinus (SSS) versus the internal jugular vein (IJV) and internal carotid artery (ICA) versus IJV correlation (Tong, Yao, et al., 2019). Their correlation coefficients, however, were much higher than ours, possibly due to their use of cross-correlation and higher temporal resolution.

A notable recent study by Huck et al. found that the intravascular venous contributions to the rs-fMRI signal decreased with increasing vascular size (Huck et al., 2023). While we did not explicitly examine the effect of diameter, qualitative examination of our data does not corroborate such a finding. For instance, although the diameter of the SSS was believed to be higher than that of the straight sinus (Larson et al., 2020), the degree and strength of connectivity from the SSS were no lower than that from straight sinus (Fig. 6). However, as we did not explicitly examine the effect of vascular diameter, we cannot conclusively comment on such differences. Nonetheless, we believe that vessel diameter is only one of the required parameters to characterize the vascular contribution to the rs-fMRI signal. In theory, an analytical model that incorporates vascular diameter, oxygenation, and orientation (relative to the main magnetic field), among other variables, can help reduce the inter-study and inter-subject variabilities in the apparent venous contribution. Moreover, based on the spatial distribution maps shown inFigures 5and6, we suggest that macrovasculature connectivity might be more susceptible to changes in orientation rather than diameter. For instance, the anterior and posterior segments of SSS, for example, had weaker strength (S_V,V_and S_V,A_) and lower degree (D_V,V_and D_V,A_) of connectivity than the superior segment. This phenomenon might be explained by different combinations of macrovasculature-related intravascular and extravascular fluctuations as described in the biophysical model of BOLD signal (Ogawa, Menon, et al., 1993).

Beyond these two previous studies, our study examined the connectivity within arterial vasculature, which also demonstrated a high degree and strength of connectivity (D_A,A_and S_A,A_). In addition, we found that correlations with a high correlation strength (e.g., S_A,A_and S_V,V_) tended to have higher correlation variance for both positive and negative correlations. This trend is also observed in spatial distributions, indicating that venous-venous correlation and arterial-arterial correlation may contribute to different RSNs to varying degrees. The macrovascular connectivity is also observable and strong at the group level, even though the extent of the effects varies among the participants.

We also noted a large amount of inter-subject variability, particularly for the degree ratio based on venous-venous correlation, arterial-venous correlation, and variance ratio based on arterial-arterial correlation. The inter-subject variability could be attributed to participants having different partial volume effects during the scan, which alter the amount of GM signal that contaminates the macrovascular signal and makes the correlation analysis less macrovascular specific. In addition, the inter-subject variability may be influenced by the brain region, which may suffer from less variability if it has a greater portion of macrovasculature. Further, the scan parameters represent potential dependencies for intersubject variability, since the temporal resolution of the imaging could affect correlation accuracy. The details of each dependency contribution would, however, be beyond the scope of this study and need future research.

### 4.3 Contribution of the macrovascular connectivity to GM functional connectivity

Macrovascular connectivity is more interpretable in the context of global connectivity. The ratios of degree of connectivity, as a more direct measurement, are meant to reflect the strength of macrovasculature connectivity in relation to macrovasculature connectivity in the GM. According to our findings, macrovascular degree of connectivity constitutes a large number of significant connections when related to whole-GM degree of connectivity (a maximum of 32.51% for D_v,v_:D_v,GM_and 6.01% for D_A,A_:D_A,GM_), even without considering the perivascular BOLD effects. Thus, when macrovasculature is included in GM ROIs, which is often the case, up to 1/3 of the significant connections could be coming from the macrovasculature. Taken together with our previous work, which showed that connectivity z-scores decreased with increasing macrovascular blood volume fraction in a number of RSNs (Tak et al., 2015), these findings indicate the macrovasculature-driven functional connectivity is indeed of concern despite their weaker connectivity strengths compared to non-macrovascular connectivity. In addition, μ(D_V,V_:D_V,GM_) was higher than μ(D_A,A_:D_A,GM_) (Fig. 8), suggesting stronger correlations between BOLD signals from veins than from arteries (Tong, Yao, et al., 2019). This finding may also be driven by the fact that veins account for a larger fraction of the GM ROI than arteries, which may also explain why μ(D_A,V_:D_A,GM_), derived from all venous voxels, is higher than μ(D_V,A_:D_V,GM_), derived from arterial voxels only.

Our findings echo findings reported byHuck et al. (2023)based on the binned values of low-frequency fluctuation amplitude and regional connectivity homogeneity, diameter, and distance metrics. However, these results are in conflict with their voxel-based modeling results using higher-order polynomials, which fit the above GM metrics (dependent variables) against venous diameter and distance from the nearest veins (as independent variables). As the goodness of fit was used to indicate the macrovascular contribution, the latter was deemed to be lower in the presence of larger diameters. One explanation for this is that the larger variability of the effects associated with larger vessels may have diminished the ability of the polynomials to fit to the connectivity metrics. Indeed, there are other biophysical parameters that would factor strongly in producing the BOLD signal especially around larger vessels, especially vessel orientation. We have previously reported on the strong influence of vessel orientation on the BOLD signal (Zhong & Chen, 2022), so we suggest that a biophysical model driven by first principles that include the relevant variables such as vascular size, oxygenation, and orientation would be needed to more fully characterize the macrovascular contribution to BOLD. Measures such as vascular position within a voxel may also strongly influence the macrovascular BOLD effect (Zhong & Chen, 2023), but are nonetheless less feasible to quantify. It is also worth noting that our results show that μ(σ^2^_A(ex),A_:σ^2^_A(ex),GM_) values are much higher than the mean of other variance ratios, suggesting that the arteries have higher inhomogeneity in terms of connectivity contribution (Fig. 9). This may be due to the smaller extent of arterial vascularity compared to venous vascularity. Furthermore, the mean variance ratios of all four correlation pairs range from approximately 0.75 to 3.5, and the histograms indicate that over half of the vascular voxels exhibit connectivity variability superior to that seen in the GM. As a result of the high variance ratios, it appears that the neural specificity of GM connectivity may be heavily impacted by whether macrovascular connectivity is included in its calculation.

Interestingly, we found that increasing the threshold for degree ratio did not necessarily result in a lower degree ratio (Supplementary Fig. S1). For example, while we increased the threshold from 0.15 to 0.5, the results from a representative subject showed the macrovasculature still had a significant influence on the correlation between vasculature and the GM. In this case, the increase in threshold may have had less effect on the connectivity between macrovasculature and GM than on the connectivity within macrovasculature.

### 4.4 Contribution of the perivascular connectivity to GM functional connectivity

Finally, we showed that the macrovascular contributions to connectivity were still evident well beyond the confines of the macrovascular space. The ratios of degree and variance were also used to determine the extent of perivascular connectivity. As seen inFigure 8, the venous-to-GM degree ratio ranged between 10% and 40% even at one voxel away from the voxel containing the vessel, while arterial degree ratios were mostly less than 6%. This is in accordance with the recent results ofHuck et al. (2023), who showed that major veins can produce a significant systemic spatial gradient across all common rs-fMRI metrics (including low-frequency fluctuation amplitude, regional connectivity inhomogeneity, Hurst exponent, and eigenvector centrality) as a function of the distance from the veins. As the distance from the macrovasculature increases, the degree ratio decreases. However, the ratio of the degree of connectivity indicated that the macrovascular effect was still detectable at three voxels away from the macrovasculature, similar to reported previously (Huck et al., 2023). Considering the resolution of the rs-fMRI scans in the Midnight Scan Club dataset is 4 mm isotropic, 3 voxels amount to 12 mm. At such a low spatial resolution, macrovascular effects are likely to be present throughout the majority of GM voxels, but at a higher resolution, 12 mm may simply translate into a 6-voxel distance, still representing a large swathe of the GM. Moreover, upon dividing the macrovasculature into veins and arteries, veins (higher D_V(ex),V_:D_V(ex),GM_) exhibited greater effects than arteries (lower D_A(ex),A_:D_A(ex),GM_). As a case in point, venous connectivity can still contribute to a group-average maximum of 30.6% of whole-GM connectivity even with 3 voxels away, but arterial perivascular effects (D_A(ex),A_:D_A(ex),GM_) are as low as 6% at just one voxel away (Fig. 10f, m). This is in keeping with the expectation that the majority of BOLD effects are driven by the venous system (Ogawa, Lee, et al., 1993;Segebarth et al., 1994).

Similar to the degree ratios, the connectivity variance ratios, which reflect spatial inhomogeneity of correlation strengths, decreased with increasing distance from vessels for both veins and arteries, signaling that spatial variability in functional connectivity may in large part be due to the macrovascular influence. The vascular variance ratios ranged from 0.5 to 1.5, as shown inFigure 11, which, in conjunction with the histograms, indicate that over half of the vascular voxels exhibit connectivity variability surpassing that typically seen in the GM. Thus, if vascular or perivascular voxels are included in regional connectivity analysis, they can potentially increase connectivity variance substantially and reduce the neuronal interpretability of the connectivity measures. Similar findings pertain to the perivascular connectivity variance, although the perivascular contribution to connectivity variance diminishes with increasing distance from the vessel (Fig. 11).

It is important to note that the spatial variance of the prevalence of perivascular effect varies widely between arteries and veins. The group-average variance ratio μ(σ^2^_V(ex),V_:σ^2^_V(ex),GM_) was 1.04, 0.914, and 0.883 at one, two, and three voxels away from macrovasculature, respectively. Conversely, μ(σ^2^_A(ex),A_:σ^2^_A(ex),GM_) was only 0.199, 0.147, and 0.136 for these distances, respectively (Fig. 11f, m). Moreover, the vascular and perivascular effect also depends on the network or region in question. The findings (Table 1) reveal that the anterior cingulate and primary visual networks contained the largest amount of macrovasculature, up to 8% even without accounting for the perivascular regions of vascular influence. Of course, the actual macrovascular contributions in these networks also depend on physical attributes of the blood vessels involved, such as vascular diameter and orientation, among others. Of note, it can be observed fromFigure 11that the orientation of the blood vessel with respect to the B0 field may also modulate the amount of connectivity variance contributed by each blood vessel. For instance, inFigure 11b, it is apparent that the superior-inferior segment of the sagittal sinus exhibited lower variance ratios than the anterior-posterior segment. However, the effect of vascular orientation is not explicitly modeled in this study, which focuses on the observable contributions. The biophysical modeling of such an effect will also need to take into account blood volume fraction and oxygenation, and will be the topic of our future work.

Two possible explanations for the above perivascular connectivity relate to magnetic susceptibility and scan technical constraints, respectively. The magnetic susceptibility difference between the blood and tissue will generate an extravascular dipolar magnetic field offset around macrovasculature, which can extend well beyond the voxel containing the vessel (Ogawa, Menon, et al., 1993). Due to such magnetic-field offsets, it is possible for voxels in the perivascular tissue to experience macrovascular-like oscillations and show a high degree of connectivity with one another. On the other hand, as the BOLD signal propagates from the macrovascular system to the medium or small blood vessels, their reduced size and flow velocities may lead to challenges in capturing them in the TOF data. In spite of the fact that these vessels exhibit similar oscillation patterns to macrovasculature, demonstrate a strong correlation with microvasculature, and might have negligible time delays (if they directly connect to large vessels), they may be “invisible” to us. It is important to note that either of these factors cannot be excluded based solely on in-vivo experimental results (both degree ratios and variance ratios), especially since these two factors may interact, and future simulation studies are necessary.

### 4.5 Recommendations

It is important to note that this study is focused only on assessing the macrovascular effects rather than correcting them or modeling them, which is the focus of our ongoing work. The key to removing macrovascular bias is to locate the macrovasculature. Therefore, we recommend that macrovascular anatomical information be collected along with rs-fMRI with either TOF imaging or susceptibility weighted imaging (SWI). At first glance, masking out the detectable macrovasculature based on angiograms may be the most straightforward method of correcting the bias. However, as we have suggested, macrovascular effect could spread well into the perivascular tissue, which makes direct masking inadequate as a remedy. Spin echo (SE) sequences rather than the conventional gradient-echo sequences have also been proposed to suppress the strong macrovascular susceptibility effects. However, this option entails a sacrifice of the signal-to-noise (Menon, 2012), and the use of EPI still renders SE sensitive to large-vein effects (Ragot & Chen, 2019). More recently, BOLD image phase was also suggested as a regressor to remove macrovascular effects in task-based fMRI (Menon, 2002;Stanley et al., 2021). The method, however, assumes that all large vessels produce a phase change and that intravascular phase offsets do not contribute to the overall signal, which may not be the case in all cases (Ogawa, Menon, et al., 1993). For example, with increasing vessel size, it becomes less appropriate to disregard the intravascular phase offset and orientation effect, as discussed in our previous work (Zhong & Chen, 2022,^2023^). Furthermore,Huck et al. (2023)suggested using high-order polynomials to model and remove both binned intravascular and binned extravascular venous bias. However, they found their model is insufficient to remove voxel-wise venous bias in rs-fMRI. Thus, the observations in this paper prompt us to consider ways of more comprehensively modeling the vascular BOLD contributions as the first step to correcting for them.

In this context, analytical modeling (Cheng et al., 2009;Ogawa, Menon, et al., 1993) is still considered to be the best method for more accurately characterizing macrovascular effects. Based on the information provided by the TOF images (fraction of blood volume and orientation of the vessels) as well as reasonable assumptions (oxygenation level), it would be possible to generate macrovascular signals using the analytical models, and use the simulated macrovascular signal to correct macrovascular biases. The extent to which such an approach can succeed is the focus of our ongoing work.

### 4.6 Study limitations

At the time of writing, the Midnight Scan Club dataset is the only public dataset that contains vascular imaging and rs-fMRI data. However, there remain limitations in the macrovascular delineation based on this data set. First, due to the limited spatial resolution of TOF images as well the large range of flow velocities across vessels of varying sizes, it is difficult to segment smaller vessels and to understand better how macrovascular factors contribute to perivascular connectivity. Secondly, the determination of arterial versus venous vessels was performed manually, allowing human error into the process, although there is no known automated alternative. Third, the spatial resolution of rs-fMRI is higher than that used in most rs-fMRI studies (Raimondo et al., 2021), so it is not yet clear to what extent our results can be directly translated into a more state-of-the-art spatial resolution. Notwithstanding, the finding of large perivascular effects outside the vascular space may well translate into a spatial-distance dependent perivascular contribution, one that is in agreement with studies at higher spatial resolutions (Huck et al., 2023). On that note, in adhering to typical preprocessing procedures, we regressed out parts of the BOLD signal in the white matter and cerebrospinal fluid, although the latter can also exhibit macrovascular contributions. Thus, these results represent a conservative estimation of the macrovascular contribution to GM rs-fMRI. Furthermore, as this study focuses on the observational perspective, the macrovascular effect is not explicitly modeled. The biophysical modelling of such an effect will also need to take into account blood volume fraction, oxygenation, and vascular orientation relative to the main magnetic field, and will be the topic of our future work.

## 5 Conclusions

To conclude, we found strong FC patterns within the macrovasculature, based on both correlations and anti-correlations (depending on the type of vasculature) at 3 Tesla. Moreover, such connectivity contributes significantly to GM FC, particularly in the vicinity of veins. Furthermore, the functional connectivity originating from the macrovasculature displayed disproportionately high spatial variability when compared to the spatial variability across all GM voxels. It is important to note that, the macrovascular contribution generally extends far beyond the confines of macrovasculature, and thus cannot be removed by simple masking. A more feasible approach may involve biophysical modeling of the macrovascular effects proximal and distal to the vasculature, which will be the focus of our future work.

## Supplementary Material

Supplementary Material

## Data Availability

All data described in this manuscript were obtained from the Midnight Scan Club (MSC) dataset. The data are available publicly at OpenNeuro. The code will be made available upon request.
