# Supplementary material for: Assessment of the macrovascular contribution to resting-state fMRI functional connectivity at 3 Tesla
Source: Imaging Neurosci (Camb). 2024 May 20;2:imag-2-00174. doi: 10.1162/imag_a_00174 (PMC12272202; doi:10.1162/imag_a_00174)
Supplement: Supplementary Material [file imag_a_00174-supp.pdf]

Appendix

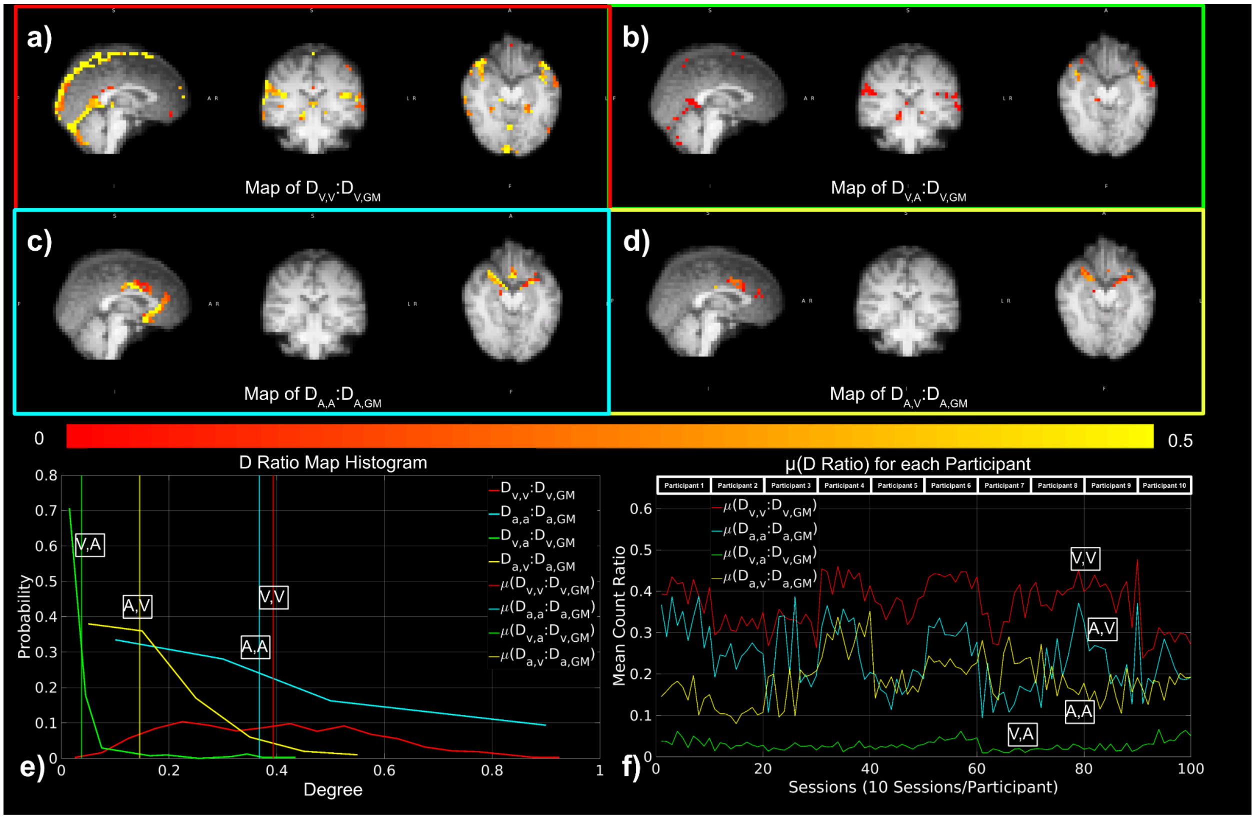

Figure S1. Contribution of macrovascular degree of connectivity to overall tissue degree of connectivity, using an elevated correlation threshold. Red indicates $D_{v,v}$:$D_{v,GM}$; yellow indicates $D_{a,v}$:$D_{a,GM}$; cyan indicates $D_{a,a}$:$D_{a,GM}$; green indicates $D_{v,a}$:$D_{v,GM}$. The results are from a representative subject as used in the previous figures, suggesting that even at a higher correlation threshold, the macrovasculature still had a significant influence on the correlation between vasculature and the GM (a-d). However, these effects were less extensive spatially compared to a lower correlation threshold (15%), especially for $D_{a,v}$:$D_{a,GM}$ and $D_{v,a}$:$D_{v,GM}$ (b,d). As shown in the histogram, although the distribution of $D_{a,a}$:$D_{a,GM}$ is broad, , the majority of degree ratios were still very low. This is not the case for $D_{v,v}$:$D_{v,GM}$ and $D_{v,a}$:$D_{v,GM}$ (e). Across all sessions and all participants, the $D_{v,v}$:$D_{v,GM}$ was the highest, while the $D_{v,a}$:$D_{v,GM}$ was the lowest (f).
